# Long-term vegetation restoration increases deep soil carbon storage in the Northern Loess Plateau

**DOI:** 10.1038/s41598-021-93157-0

**Published:** 2021-07-02

**Authors:** Zhilong Lan, Ying Zhao, Jianguo Zhang, Rui Jiao, Muhammad Numan Khan, Tanveer Ali Sial, Bingcheng Si

**Affiliations:** 1 College of Resources and Environmental Engineering, Ludong University, YANTAI, 264025 China; 2 Key Laboratory of Plant Nutrition and the Agri‑Environment in Northwest China, Ministry of Agriculture, College of Natural Resources and Environment, Northwest A&F University, Yangling, 712100 China; 3 State Key Laboratory of Soil Erosion and Dryland Farming On the Loess Plateau, Northwest A&F University, Yangling, 712100 China

**Keywords:** Biogeochemistry, Environmental sciences

## Abstract

Afforestation plays an important role in soil carbon storage and water balance. However, there is a lack of information on deep soil carbon and water storage. The study investigates the effect of returning farmland to the forest on soil carbon accumulation and soil water consumption in 20-m deep soil profile in the hilly and gully region of the Chinese Loess Plateau. Four sampling sites were selected: *Platycladus orientalis* (Linn.) Franco forest (PO: oriental arborvitae), *Pinus tabulaeformis* Carr. Forest (PT: southern Chinese pine), apple orchard (AO) and farmland (FL, as a control). Soil organic carbon (SOC) and soil inorganic carbon (SIC) content were measured in 50-cm sampling intervals of 20-m soil profiles, as well as the associated factors (e.g. soil water content). The mean SOC content of PT was the highest in the 1–5 m layer and that of FL was the lowest (*p* < 0.05). Compared with FL, the SOC storages of PO, PT and AO increased by 2.20, 6.33 and 0.90 kg m^−2^ (*p* > 0.05), respectively, in the whole profile. The SIC content was relatively uniform throughout the profile at all land-use types and SIC storage was 9–10 times higher than SOC storage. The soil water storage of PO, PT and AO was significantly different from that of FL with a decrease of 1169.32, 1161.60 and 1139.63 mm, respectively. After the 36-yrs implementation of the “Grain for Green” Project, SOC in 20 m soil profiles increased as a water depletion cost compared with FL. Further investigation is still needed to understand the deep soil water and carbon interactions regarding ecological restoration sustainability in the Northern Loess Plateau.

## Introduction

Environmental problems, such as soil degradation and global climate change, have received increasing attention in the last few decades^1–3^. Afforestation is very often used to improve soil degradation for increasing carbon sequestration and mitigating atmospheric CO_2_ concentration^2,3^. Carbon distribution and water balance are vital components for the sustainable development of terrestrial ecosystems in the revegetation processes^4,5^.

The soil carbon pool includes soil organic carbon (SOC) and soil inorganic carbon (SIC) pool. The soil carbon storage in the top 1 m layer is around four times the vegetation carbon pool and three times the atmospheric carbon pool^3,6^. Notably, even small changes in the soil carbon pool can significantly influence the atmospheric CO_2_ concentration^7^. However, the number of existing studies on deep soil carbon storage is limited. According to Jobbagy and Jackson^8^, the average global SOC storage in the 0–3 m soil layer is 56% higher than that in the 0–1 m layer. Fearnside and Barbosa^9^ reported that out of the carbon storage in the 0–8 m soil profile in Amazonia (136 Gt), only 34.56% (47 Gt) is found in the 0–1 m soil layer. Liu et al.^10^, investigating the gully region of the Chinese Loess Plateau, discovered that SIC storage in the 0.4–2 m layer represented around 85% of the total SIC storage in the 0–2 m layer, implying that massive amounts of SIC may have stored in the relatively deep soil. Meanwhile, revegetation consumed more than 1500 mm soil water storage (SWS) from the deep soil profile compared with cropland^5,11^. Expectedly, implementing the “Grain for Green” Project-one of the most extensive ecological programs in China on converting cropland to the forest, has had a considerable effect on the SOC, SIC storage and SWS in the Chinese Loess Plateau^10–13^.

Previous studies have unanimously reported that ecological restoration increased SOC storage in the top layer (0–1 m) and decreased SWS^10,14^; however, similar investigation concerning SIC has been lacking^15,16^. SIC is also an essential part of the soil carbon pool, mainly as carbonate in arid and semiarid regions, where its content has been reported to be 5–10 times that of SOC^12,17^. In China, the north-western arid and semiarid regions were considered to represent 60% of the SIC of the country^12^, and were a potentially large carbon pool^18^, which could play a vital role in alleviating the increasing atmospheric CO_2_ concentration in the global carbon cycle^19^. Previous research showed that carbon in deep soil is more stable and provides long-term storage, which may play a more critical role in soil carbon sequestration^20,21^. However, it is not clear how much SOC and SIC storage in a deep soil and the relationship of SOC and SIC storage with SWS expense below 1 m in the Chinese Loess Plateau^22^.

Nowadays, the ecological environment of the Chinese Loess Plateau has been dramatically improved with the natural afforestation and tree plantation^12,23^. The afforestation of previously cultivated land is potentially considered to sequester carbon into the soil^4,24^; however, previous research was mainly focused on shallow 0–2 m soil profile regarding the effect of afforestation on the SOC content, despite the importance of SOC and SIC storage at depths below 2 m^23,25,26^. Accurate estimation is still challenging due to a lack of information on SOC and SIC and the difficulty of sampling deep soil^27^. Previous studies were reported that different vegetation species and restoration age might have significantly affected SOC capacity; however, the changes in the SIC following afforestation have not been well detected in the Chinese Loess Plateau^14,16^. Revegetation in the Chinese Loess Plateau has remarkably influenced the soil carbon and water processes^22,27,28^. It is reported that afforestation in the Loess Plateau is approaching sustainable water resource-use limits and near the maximum potential for carbon sequestration, thus threatening local/regional water and ecological security^22^. Moreover, although few studies have focused on understanding how land-use change affects water and carbon dynamics, most of these efforts have focused on shallow soil^29^. Zhao et al.^30^ have reported negative correlations between SOC and SIC in the 0–50 cm soil under forestland, shrubland, and grassland. However, the relationship between SOC and SIC storage with SWS among different tree plantation types remains unclear, particularly how deep soil carbon and water interact. In deep-rooted ecosystems, sampling at shallow depths results in underestimating soil carbon stocks, and this inadequate result has misled our understanding of the co-evolution and interaction of deep soil water and carbon following afforestation^29^.

This study sought to explore the effects of different vegetation types on deep SOC, SIC and soil water content to provide a rational basis for sustainable afforestation in the hilly and gully region of the Northern Loess Plateau. The objectives of the study were (1) to evaluate the effects of afforestation types on both SOC and SIC distribution; (2) to quantify the change of deep (0–20 m) vs. shallow (0–1 m) soil carbon storage following the afforestation of cropland; and (3) to understand the coupling interactions between deep soil carbon storage and SWS below 1 m in the different revegetation types.

## Materials and methods

### Study area

**Figure 1:**
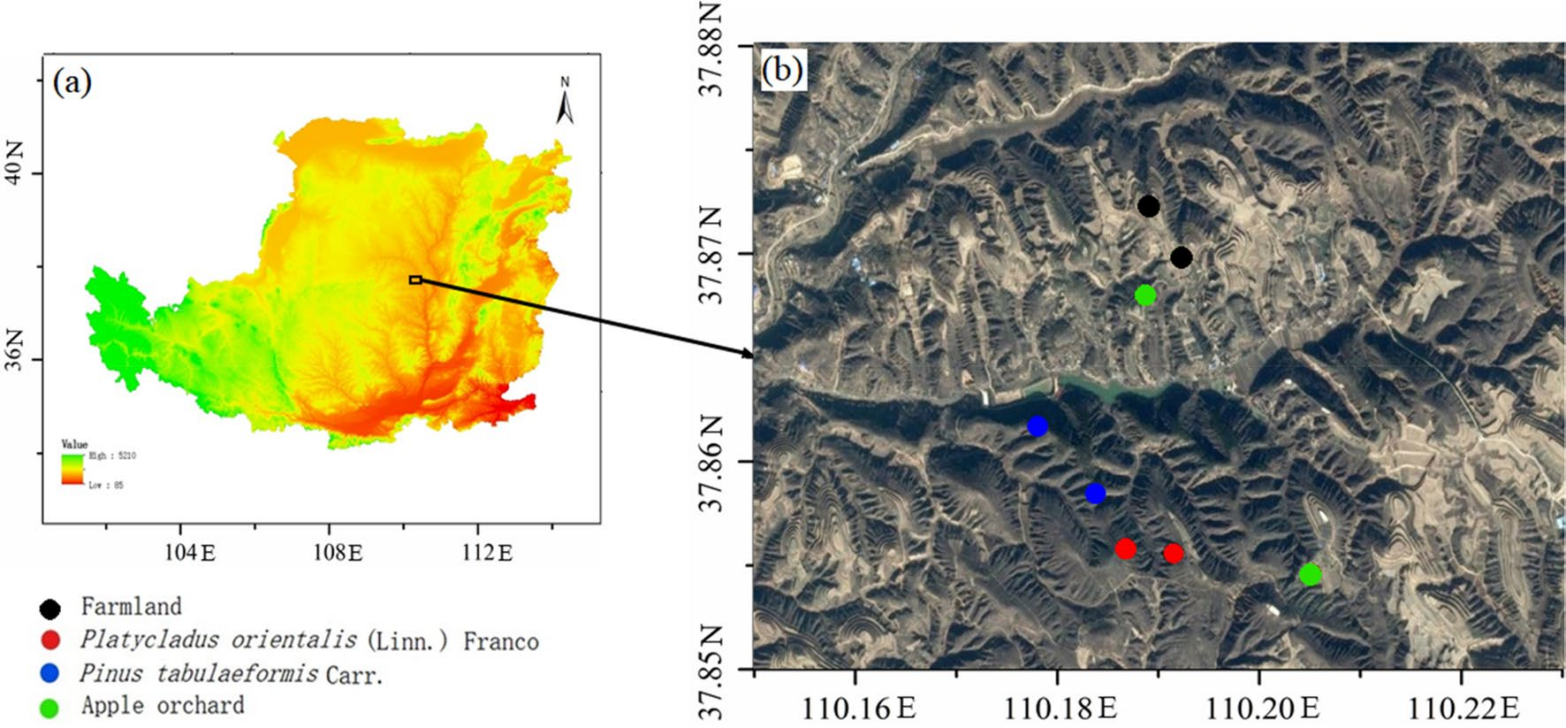
Distribution of the sampling sites in the study area (map created using ArcGIS 10.2 software by first author, http://www.esri.com).

The study was carried out at Gaoxigou Village (37° 87′ N, 110° 18′ E), Mizhi County, Shaanxi Province, an ideal research site characterized by a distinct Chinese national demonstration area soil and water conservation. It is possibly one of the most extended and well-preserved regions with a clear history of relatively diversified land management, as well as the remarkable contribution of the “Grain for Green” Project in the north of the Chinese Loess Plateau (Fig. 1). The region has a semi-arid continental climate, with a mean annual temperature of 8.4 °C and mean annual precipitation of 440 mm, about 75% concentrated in July to September. The groundwater level is at more than 50 m depth. The region has a typical hilly and gully topography with an elevation of 1000–1100 m (a.s.l.), and a loessial soil developed on the loess parent material classified as the Loessi-Orthic Primosols (Chinese Soil Taxonomy). Several types of forest have been planted on former arable land, including *Platycladus orientalis* (Linn.) Franco (oriental arborvitae) and *Pinus tabulaeformis* Carr. (southern Chinese pine), as well as various orchards.

### Experimental design and soil sampling

Three forestry types: *Platycladus orientalis* (Linn.) Franco forest (PO), *Pinus tabulaeformis* Carr. forest (PT) and apple orchard (AO), as well as one farmland (FL) as a control, were selected in this study, and two sites (spatial independent; Fig. 1) were sampled per vegetation type. We focused on the recent decadal revegetation effects on soil carbon accumulation using a chronosequence approach. Before 1979, all three selected forestry sites were arable land, which was then returned to the forest, while the FL site remained as arable land, primarily planted with potatoes (*Solanum tuberosum* L.), green onions (*Allium fistulosum* Linn.) and millet (*Setaria italic* (L.) Beauv.). Mean annual nitrogen (N), phosphorus (P), and potassium (K) fertilizer applications were 180, 60, and 75 kg ha^−1^, respectively, for farmland. Mean annual nitrogen (N), phosphorus (P), and potassium (K) fertilizer applications were 650, 300, and 200 kg ha^−1^, respectively, for the apple orchard. Historically, the soil characteristics and management (tillage and fertilizer application) practices are mostly uniform in this region, and intensive agricultural practices peaked in the 1970s^22^. Therefore, it is assumed that there were no differences in the soil physicochemical properties between the forest and farmland sites before tree plantation in 1979 because of the long-term cultivation at all sites before this date. After the farmland conversion to a tree plantation, there was basically no human activity except for the apple orchard. Each pair of sites (i.e. two replicates, 30 m × 30 m quadrat) had similar topography (slope direction, degree and landform position). For each pair of sites, the vegetation species composition was similar and the vegetation cover was about 70–80% in all sites (Table 1).

**Table 1:** Basic characteristics of the experimental sites (mean ± SD, n = 2).

Type	Vegetation height (m)	Landform position	Slope direction	Slope degree (°)	Altitude (m)
FL	0.3 ± 0.1	Terrace	Southeast	–	1069
PO	3 ± 0.5	Middle slope	Southeast	12^°^	1076
PT	5 ± 0.9	Middle slope	Southeast	10^°^	1018
AO	3.5 ± 0.7	Terrace	East	–	1084

*FL* farmland, *PO* *Platycladus orientalis* (Linn.) Franco, *PT* *Pinus tabulaeformis* Carr., *AO* apple orchard, – data not available.

Soil samples were collected from the selected sites (Fig. 1) in July 2015. To minimize the influence of climate, the sampling was taking during the rainfall free period. Soil samples were collected using a soil auger down to 20 m (except for one of the two PO and AO profiles, which were sampled to 17.6 m and 15.4 m, respectively, due to geological restrictions). Each soil sample constitutes a mixture of a 50 cm soil layer collected manually with a 6 cm diameter auger. Thus, 31–41 samples in total were collected from each of the 8 profiles.

### Laboratory analysis

All samples were air-dried for 7 days in the laboratory and then passed through a 2-mm sieve to remove stones, roots, and other debris for further analysis. SOC content was determined using the potassium dichromate oxidation method with external heating^31^. SIC content was determined using the gas volume method^32^. To determine the soil bulk density (γ_i_), undisturbed soils in the middle part of the soil auger were collected (100 cm^3^). Samples for soil water content (20 cm interval) and bulk density measurements (50 cm interval) were weighed before and after drying at 105 °C for 12 h to a constant mass. Soil particle composition analyses by pipette sampling method and the vertical distribution of soil sand, silt and clay content shown in Fig S1. The roots were also collected using a root auger (diameter 6 cm) layer by layer (20 cm interval), washed with tap water to remove soil particles, placed in a plexiglass tray with 1 cm water, and then scanned using the EPSON Perfection V700. The root densities were analyzed in WinRHIZO software (Regent Instruments Inc.).

The storages of SOC and SIC were calculated using the following formula:

1 $$SOCS_{i} \left( {or{\text{ }}SICS_{i} } \right) = \frac{{\left( {1 - \delta \% } \right) \cdot C_{i} \cdot h \cdot \mathop \gamma \nolimits_{i} }}{{100}}$$

where *SOCS*_*i*_ (kg m^−2^) is the soil organic carbon storage of the *i*th layer, *SICS*_*i*_ (kg m^−2^) is the soil inorganic carbon storage of the *i*th layer, *δ* (%) is the gravel content (the gravel content of the samples was 0), *C*_*i*_ (g kg^−1^) is SOC or SIC content, *h* (cm) is the thickness of soil layer, and 100 is the unit conversion coefficient. *γ*_*i*_ (g cm^−3^) is the soil bulk density calculated as follows:

2 $$\mathop \gamma \nolimits_{{i,i + 50}} = \frac{{{\text{m}}_{i} }}{{(1 + {{\omega _{i} } \mathord{\left/ {\vphantom {{\omega _{i} } {100}}} \right. \kern-\nulldelimiterspace} {100}}) \cdot V}}$$

where *ω*_*i*_ (%) is the soil gravimetric water content of each layer, *m*_*i*_ (g) is the weight of fresh soil in the *i*th layer and *V* (cm^3^) is the soil volume.

SWS was calculated using the following formula:

3 $$SWS_{i} = \frac{{\omega _{i} \cdot h \cdot \mathop \gamma \nolimits_{i} }}{{\rho \cdot {\text{10}}}}$$

4 $${\text{SWS loss}} = \sum\nolimits_{{i = 1}}^{n} {SWS_{{Ai}} } - \sum\nolimits_{{i = 1}}^{n} {SWS_{{Fi}} }$$

where *SWS*_*i*_ (mm) is the soil water storage of the *i*th layer, *ρ* (g cm^−3^) is water density, 10 is the unit conversion coefficient, *SWS*_*Ai*_ (mm) is the soil water storage of forestry types, *SWS*_*Fi*_ (mm) is the soil water storage of FL and the other variables are describing in Eqs. (1) and (2).

### Statistical analysis

SPSS 16.0 (SPSS Inc., Chicago, IL, USA) statistical software was used for the statistical analysis. Revegetation type and soil layers were considered as the main effects. Based on the averaged soil water content values, root distribution, and variation coefficients, we generally divided the soil profiles into four layers: 0–1, 1–5, 5–10 and 10–20 m. Based on Li et al.^11^, rainfall infiltration depth is usually below 1 m, and the maximum depth can not exceed 5 m; we consider our depth classification is reasonable. Analyses of variance were performed using the one-way ANOVAs procedure in SPSS. The Duncan method (*p* < 0.05) assessed the differences in SOC and SIC content. Relationships between soil carbon content and basic soil properties were quantified using Pearson’s correlation coefficient. Data for soil at a depth > 1.0 m were used for correlation analysis of SOC and SIC storage with SWS because the 0–1.0 m layer could be affected by short-term rainfall dynamics.

### Ethics approval

We confirm that all experiments were performed in accordance with relevant named guidelines and regulations. We have obtained the proper permission to collect samples of plant roots.

## Results

### Vertical distributions of SOC and SIC for different vegetation types

**Figure 2:**
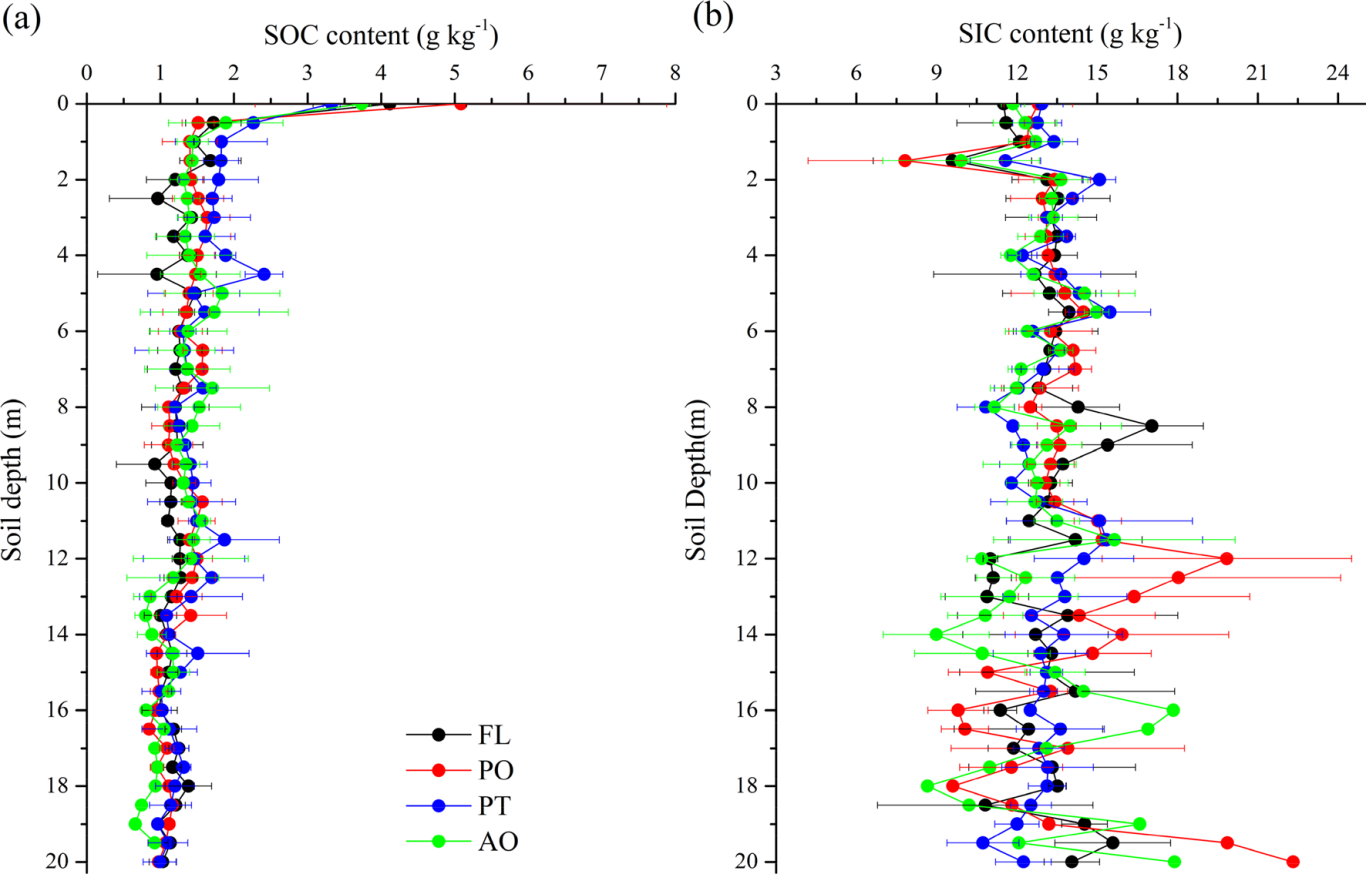
Vertical distributions of SOC and SIC content for different vegetation types. *FL* farmland, *PO* *Platycladus orientalis* (Linn.) Franco, *PT* *Pinus tabulaeformis* Carr., *AO* apple orchard. Bars are standard deviations.

**Figure 3:**
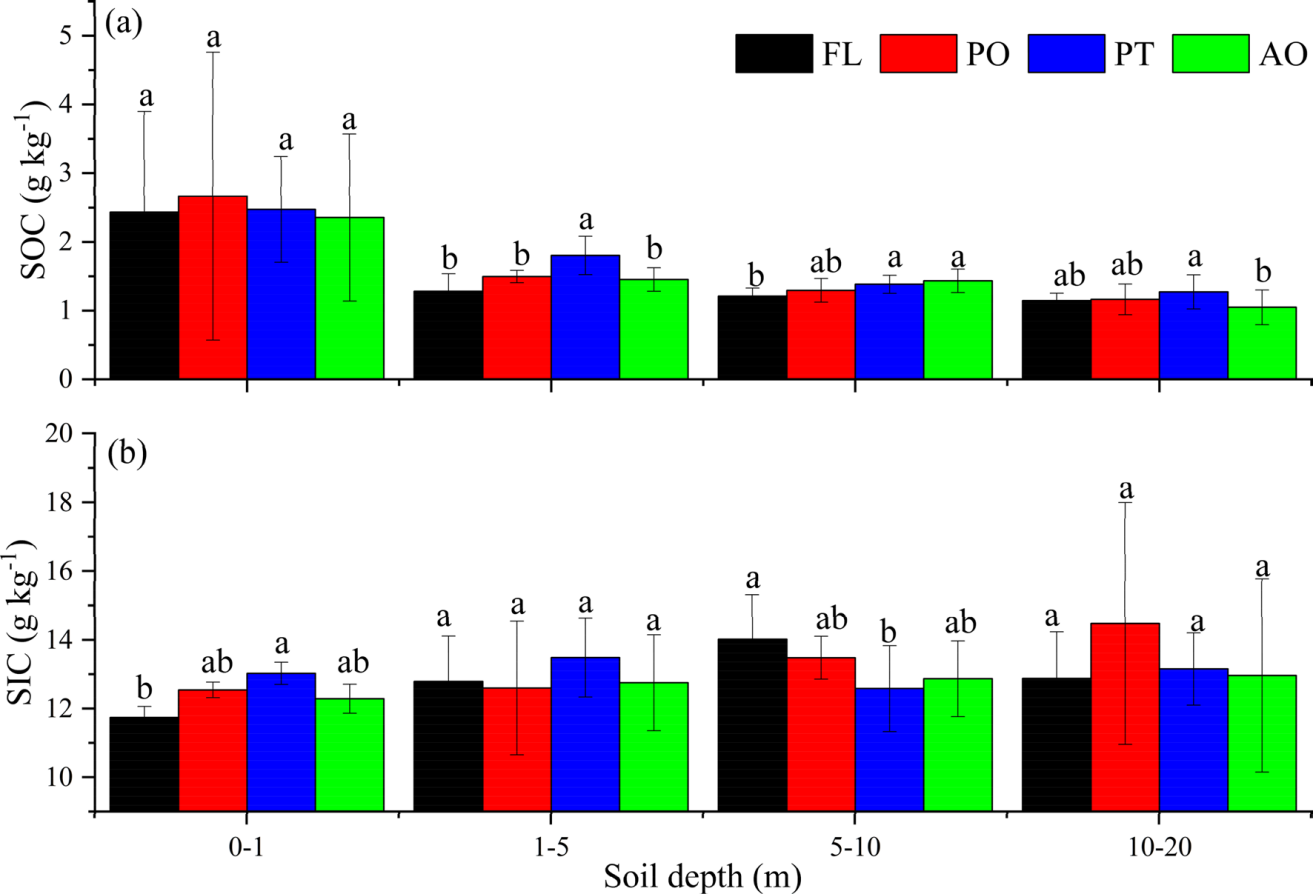
Average SOC (**a**) and SIC (**b**) content in 0–1 m, 1–5 m, 5–10 m and 10–20 m soil layers for different vegetation types. *FL* farmland, *PO* *Platycladus orientalis* (Linn.) Franco, *PT* *Pinus tabulaeformis* Carr., *AO* apple orchard. Bars are standard deviations. Different lower-case letters indicate significant differences at *p* < 0.05 among vegetation types within each layer.

In general, the vertical distributions of SOC and SIC vary with vegetation types and soil depths (Fig. 2). SOC content showed an overall downward trend with soil depth (Fig. 2a). SOC content was the highest in the top layer, then decreasing markedly throughout a 1-m soil profile. The SOC content of the 1–20 m soil layer remained the relatively small fluctuations at each site (Fig. 2). The SOC content of different land uses showed a lower value in the 10–20 m soil layer. There were no significant differences between FL, PO, PT, and AO in the 0–1 m layer (Fig. 3a). The mean SOC content of PT was higher than other land uses in the 1–5 m layer, SOC content of FL was lower than PT and AO in the 5–10 m layer, and SOC content of PT was significantly different from AO in the 10–20 m layer (Fig. 3a).

SIC content in the 0–20 m soil profile showed lower values within the 1–2 m soil layer than the layers below 2 m (Fig. 2b), with significant differences in the 0–1 and 5–10 m layers between FL and PT (Fig. 3b). For the topsoil layer, the SIC content for the various vegetation types differed and reached the lowest values at a depth of 1.5 m. In the 2–12 m soil layer, SIC content fluctuated slightly (Fig. 2b), with the highest value in PT (19.85 g kg^−1^) and the lowest value in AO (9.61 g kg^−1^). In contrast, the distribution of SIC changed sharply and irregularly at 12–20 m, notably demonstrated greater variations at PT and AO sites.

### Soil carbon storage and soil water storage

**Figure 4:**
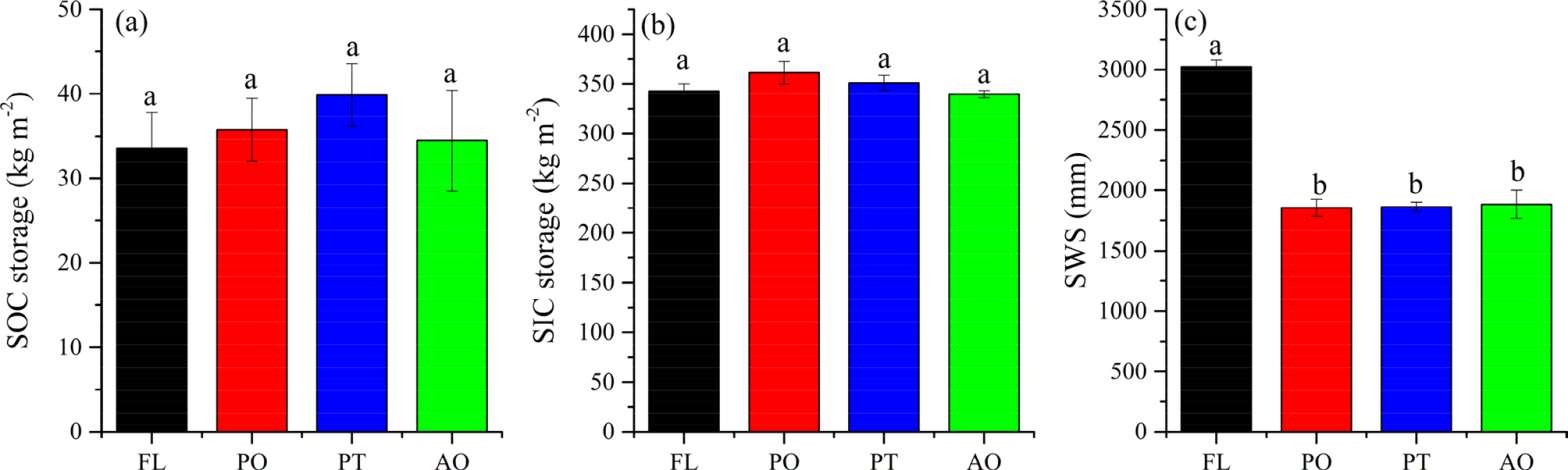
SOC storage (**a**), SIC storage (**b**) and SWS (c) in 0–20 m soil layer at all treatments. *FL* farmland, *PO* *Platycladus orientalis* (Linn.) Franco, *PT* *Pinus tabulaeformis* Carr., *AO* apple orchard. Bars are standard deviations.

**Figure 5:**
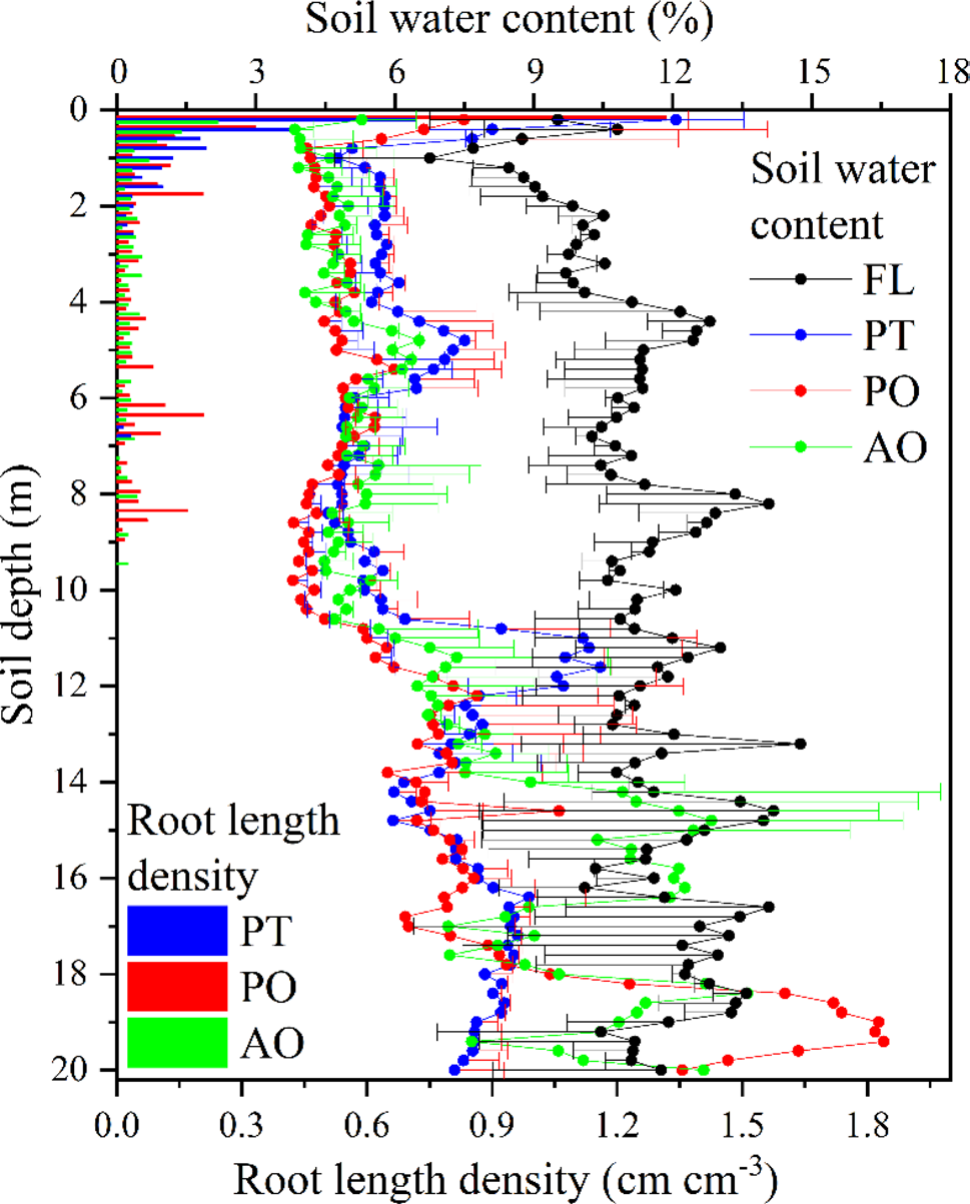
Vertical distributions of root length density and soil water content for different vegetation types. *FL* farmland, *PO* *Platycladus orientalis* (Linn.) Franco, *PT* *Pinus tabulaeformis* Carr., *AO* apple orchard. Bars are standard deviations.

Table 2 indicates that SIC storage was far higher than SOC storage in each layer under different land uses. The total soil carbon storage of FL, PO, PT and AO in the 0–20 m profile was 376.38, 396.86, 390.73 and 374.01 kg m^−2^, respectively. SOC and SIC storages within different soil layers were not significantly different among land uses (Table 2, Fig. 4). In contrast, SWS within 1–5, 5–10 and 0–20 m showed a significant difference between FL and other land uses (Table 2, Fig. 4). In the revegetation sites, soil water content within 1–11 m was smaller than FL (Fig. 5, *p* < 0.05). SOC storages in the top layer (0–0.5 m) were significantly higher than below the 0.5 m soil layer (Table S3); however, it was not significant among different land uses within different soil layers (0–2.0 m).

**Table 2:** Soil organic carbon (SOC) storage, soil inorganic carbon (SIC) storage and soil water storage (SWS) (mean ± SD, n = 2) of different soil layers for different vegetation types.

	Layer/m	FL	PO	PT	AO
SOC storage/kg m^−2^	0–1	3.56 ± 0.42a	3.98 ± 1.82a	3.48 ± 0.03a	3.25 ± 0.52a
	1–5	7.37 ± 1.10a	8.24 ± 1.32a	10.55 ± 2.33a	8.24 ± 1.73a
	5–10	9.10 ± 0.20a	9.04 ± 0.32a	11.09 ± 0.95a	10.58 ± 1.10a
	10–20	15.52 ± 0.31a	15.80 ± 1.00a	17.87 ± 2.04a	15.59 ± 1.40a
	0–20	33.54 ± 4.26a	35.74 ± 3.74a	39.87 ± 3.68a	34.44 ± 5.97a
SIC storage/kg m^−2^	0–1	13.95 ± 1.87a	15.12 ± 0.68a	16.03 ± 1.07a	13.95 ± 0.44a
	1–5	72.41 ± 6.03a	69.89 ± 1.21a	78.82 ± 2.48a	72.31 ± 1.99a
	5–10	87.56 ± 7.02a	85.74 ± 2.32a	85.74 ± 2.32a	82.35 ± 6.33a
	10–20	168.51 ± 5.74a	175.13 ± 6.59a	175.13 ± 6.59a	164.99 ± 7.98a
	0–20	342.84 ± 7.20a	361.12 ± 11.45a	350.86 ± 7.60a	339.57 ± 3.74a
SWS/mm	0–1	11.05 ± 2.35a	7.92 ± 4.73a	12.29 ± 2.64a	5.34 ± 0.02a
	1–5	60.09 ± 3.61a	26.29 ± 1.80b	35.30 ± 0.40b	28.12 ± 3.01b
	5–10	72.61 ± 10.01a	28.69 ± 1.03b	38.20 ± 5.06b	33.70 ± 3.22b
	10–20	145.67 ± 15.77a	85.80 ± 80.96a	88.11 ± 16.33a	87.36 ± 36.08a
	0–20	302.44 ± 5.46a	185.51 ± 7.12b	186.28 ± 3.99b	188.44 ± 11.67b

Different upper and lower-case letters indicate significant differences at *p*< 0.05 among vegetation types and averaged layers, respectively.

*FL* farmland, *PO* *Platycladus orientalis* (Linn.) Franco, *PT* *Pinus tabulaeformis* Carr., *AO* apple orchard.

The SIC storages of the 20-m profile for FL, PO, PT and AO were 10.22, 10.11, 8.80 and 10.05 times the SOC storages, respectively. As shown in Fig. 4, the SOC storages of PO, PT, and AO were 2.2, 6.33 and 0.90 kg m^−2^ higher than that for FL, respectively (*p* > 0.05). The SIC storage was 18.28 and 8.02 kg m^−2^ higher at PO and PT than for FL (*p* > 0.05). The SWS of PO, PT, and AO was significantly different from that of FL (Table 2), decreasing 1169.32, 1161.60 and 1139.63 mm, respectively (Fig. 4).

### Relationships between soil carbon and water storage

**Figure 6:**
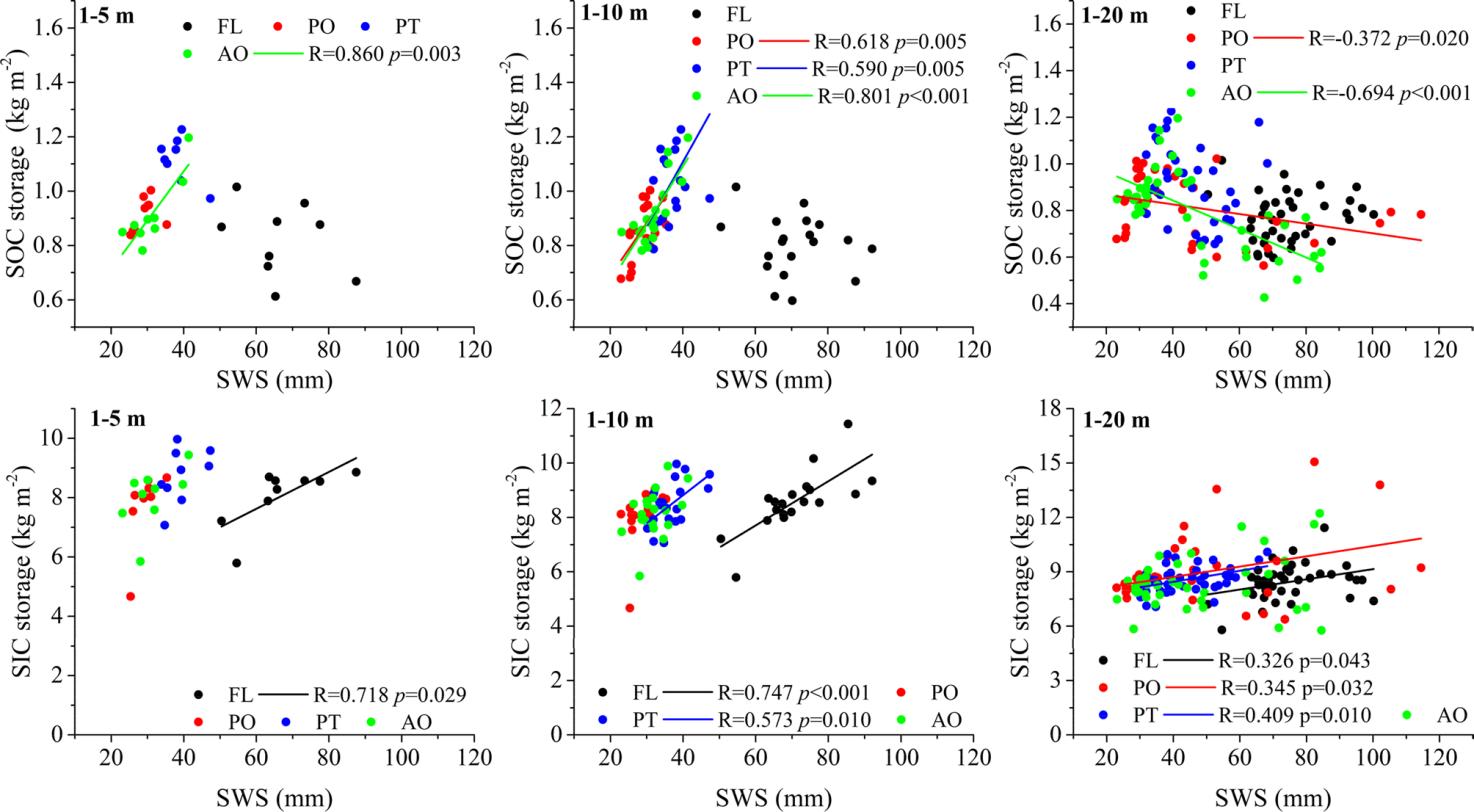
Relationships between SOC storage and SWS, SIC storage and SWS in the 1–5 m, 1–10 m and 1–20 m soil layers for different vegetation types. *FL* farmland, *PO* *Platycladus orientalis* (Linn.) Franco, *PT* *Pinus tabulaeformis* Carr., *AO* apple orchard.The only regression lines with statistical significance (*p* < 0.05) were drawn in the figure.

As shown in Fig. 6, SOC and SIC storage up to different soil depths present different correlations with SWS. In the 1–5 m soil layer, SOC storage is positively correlated with SWS in PO, PT, and AO but negatively correlated in FL, and only the AO has a significant correlation (*p* < 0.01). In the 1–10 m layer, there are significant positive correlations between SOC storage and SWS at all three revegetation types (*p* < 0.01, Fig. 6). Root length density gradually decreases at a 0–2 m soil profile at all sites and keeps stable and small values below the 2-m soil depth, which matches very well with profiled soil water content (Fig. 5). There is no root detected below 10 m soil depth for all revegetation types and the whole soil profile for the FL during the sampling period. However, in the 1–20 m layer, SOC storage and SWS are negatively correlated for all land uses, with significant correlations (*p* < 0.05) at PO and AO sites (Fig. 6).

**Figure 7:**
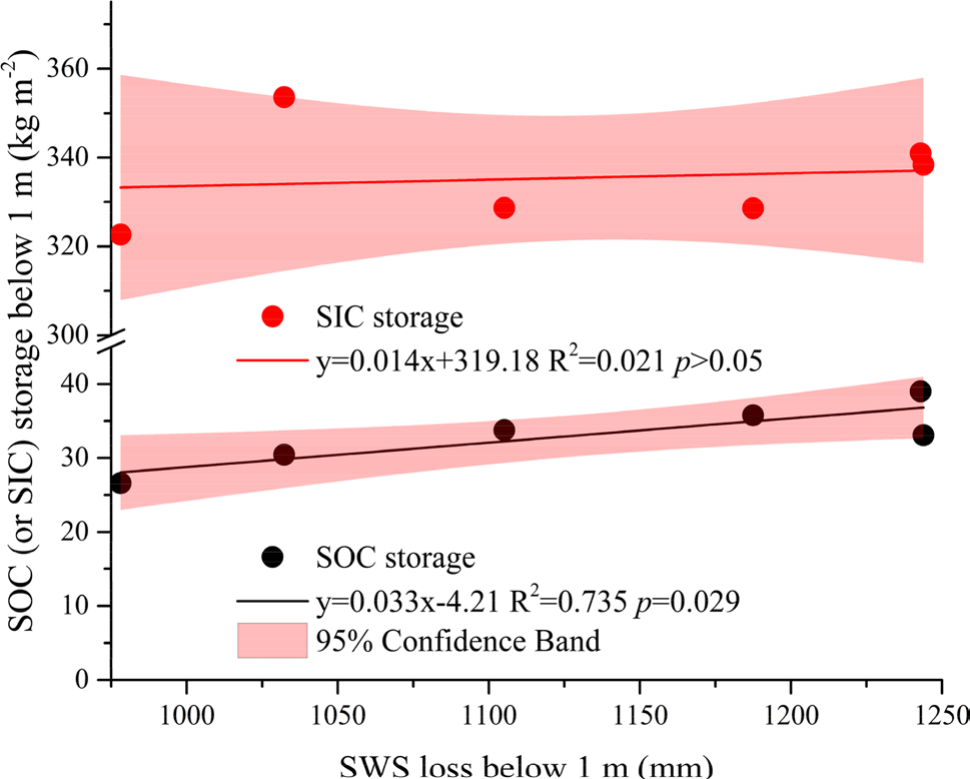
Relationship between SOC and SIC storage with SWS loss below 1 m down to 20 m.

SIC storage is positively correlated with SWS for all land uses for the 1–5 m layer, with only a significant correlation (*p* < 0.05) for FL (Fig. 6). For the 1–10 m layer, SIC storage present significant positive correlations with SWS for FL and PT, but no significant correlation for PO and AO (*p* > 0.05). There were significant positive correlations in the 1–20 m layers for all sites, except for AO (*p* < 0.05). Interestingly, for the farmland, while there is no correlation between SOC storage and SWS regardless of soil depth classifications, a significant correlation between SIC storage and SWS found for all soil depth classifications (Fig. 6). Overall, Table 3 also shows a significant negative correlation between SOC and BD for all three forestation types, but not for the farmland. In contrast, there is no clear correlation between SIC and BD or other soil chemical and physical properties (Table 3). However, soil pH shows a significantly positive correlation with SIC at PT and PO (Table 3, *p* < 0.01). Note that there is no correlation between SOC and SIC, regardless of land-use types. Moreover, SOC storage below 1 m was significantly correlated with SWS loss below 1 m (R^2^ = 0.735, Fig. 7).

**Table 3:** Pearson correlation analyses of soil properties and soil carbon under different treatments.

Treatments	Carbon type	BD	pH	Sand	Slit	Clay	Root density	SOC
FL	SOC	− 0.183	− 0.204	0.476**	− 0.517**	− 0.176	–	1.000
	SIC	0.048	− 0.055	− 0.057	0.099	− 0.056	–	− 0.280
PO	SOC	− 0.342*	− 0.457**	− 0.037	0.316*	− 0.352*	0.975**	1.000
	SIC	0.278	0.480**	0.264	− 0.295	0.111	− 0.078	− 0.115
PT	SOC	− 0.527**	0.094	− 0.437**	0.228	0.444**	0.964**	1.000
	SIC	− 0.114	0.317*	− 0.215	0.082	0.437**	− 0.263	0.019
AO	SOC	− 0.594**	0.034	− 0.021	0.196	− 0.425**	0.908**	1.000
	SIC	0.220	0.170	0.162	− 0.069	− 0.243	− 0.196	− 0.040

*BD* bulk density, *SOC* soil organic carbon, *SIC* soil inorganic carbon, *FL* farmland, *PO* *Platycladus orientalis* (Linn.) Franco, *PT* *Pinus tabulaeformis* Carr. , *AO* apple orchard.

*p < 0.05; **p < 0.01; “–” no data available.

## Discussion

### Effects of afforestation on SOC

Along with afforestation, changes in the plant species composition can alter litter input and root distribution^33^; thus, the distribution of SOC in the soil profile is changed. Vegetation patterns in the hilly and gully region of the Chinese Loess Plateau have been changed dramatically since implementing “Grain for Green” Project. The increased soil carbon content of the reforested area from farmland at the topsoil (0–1 or 2 m) has been widely reported^13,21^. Like many other studies, the SOC for all land uses demonstrated a decreasing trend with soil depth^21,28,34^. SOC content predominantly depends on the input, decomposition, and transformation of organic matter in different soil layers^20^. The SOC of the topsoil layer mainly comes from the accumulation and decomposition of external litter and root exudates^21,35,36^. With an increase in soil depth, the roots of vegetation gradually decrease, which has resulted in a decrease of SOC, as evidenced by a very significant positive correlation between SOC and plant root density (Table 3).

Our study identified that the conversion of farmland to forest slightly increased SOC content in the 1–5 and 5–10 m soil layers (Fig. 3, Table 2), but afforestation did not significantly increase SOC content compared with FL 0–1 m soil layer. The SOC in the top layer (0–1 m), active roots layer (1–10 m) and 10–20 m layer mainly affected by the management measures (e.g. tillage and remove litters), the distribution of roots, soil sedimentary process and soil texture in the Chinese Loess Plateau^9,14,17^, respectively. The variation of SOC was relatively small due to (1) SOC content of the PO was lower than that of PT due to the species-dependent root traits^24^ and shallow root distribution (Fig. 5), (2) coniferous (PT) have a limited capacity to improve soil carbon storage after restoration, and (3) the limited sample size results in the statistical analysis was difficult. In addition, the SOC did not significantly increase in all forestry types in the deep soil depth maybe because there was lower root biomass per unit soil depth and soil water consumption to wilting point limits the productions of root litter^29^. In contrast, AO had reduced SOC on the top (0–0.5 m) presumably due to management measures, such as deep plowing and loose soil, which accelerate the mineralization decomposition of SOC, but low SOC at FL was due to remove aboveground litter decreased organic matter input, and deep plowing increased soil organic matter decomposition reduced SOC accumulation^37^.

### Effects of afforestation on SIC

SIC represents the largest soil carbon pool in the Chinese Loess Plateau, mainly related to the parent material's lithology and the paleoclimate. The parent material in this study area is loess, which contains many lithogenic carbonates^38^ and its SIC content is much higher than SOC content^17,36^. Our result agreed with previous studies that SIC content for all land uses increased in the 0–1 m soil layer^10,16^, which may be due to precipitation can dissolve atmospheric CO_2_ and form H_2_CO_3_, which may react with CaCO_3_ in the soil to form soluble Ca(HCO_3_)_2_ and then leach downwards through precipitation to accumulate in the deeper soil layer. Besides, rich-organic acids in the surface soil can dissolve some soil carbonate, reducing the surface SIC^38^. In the 1.5 m soil layer, the SIC was significantly decreased in all land use (Fig. 2) due to the red clay sequence in this layer compared with other layers, where the Fe_2_O_3_ ratios generally remain high^39^ as the climate was humid during that period. Thus, the variation of SIC has fluctuation at the depth 2–10 m slightly and great fluctuation at the depth > 10 m, which was explained by that the soil lithogenic carbonates and paleoclimate play a vital role in SIC distribution at the depth 2–10 m, and is likely caused by soil-forming processes (loess parent material and deposition process), large spatial variability or the paleoclimate at the depth > 10 m^39–41^.

The variation of SIC content for the various vegetation types were increased with the increasing at 0–1 m soil layer and SIC in PO and PT were relatively higher than in FL and AO in the 0–0.5 m, which was explained by (1) the nitrogen fertilizer application may have induced the soil carbonate reduction forming CO_2_ throughout soil profile to the atmosphere^36,42,43^, (2) tillage increase precipitation infiltration at the terraced FL and AO thus induce the leaching SIC from top (0–0.5 m) soil layer, and (3) organic acid and amino acid contained in root exudates at PO and PT sites reduce the SIC content^10^. For this reason, SIC at all sites is slightly higher below 2 m than the above due to the carbonate deposition and paleoclimate. It is suggested that the paleoclimate role at this layer is the main reason for the difference^36,39^; the effect of vegetation type on soil inorganic carbon in deep soil is weak. Chang et al.^15^ examined that land-use conversion from cropland to forest could redistribute SIC along with the soil profile but would not affect the net SIC accumulation.

### Implications of revegetation on soil carbon and water storage

This study discovered that the SOC and SIC storage of the whole soil profile (0–20 m) was 9 and 24 times that of the topsoil layer (0–1 m), respectively. The results indicated that the study area stores large amounts of SOC and SIC below the topsoil profile. Furthermore, since SIC storage is a substantial component, 9–10 times SOC storage in the 0–20 m soil profiles for different land uses (Fig. 4a,b). Therefore, soil carbon storage might have been greatly underestimated in this area and other similar regions^9,44,45^. Soil carbon accumulation in deep (below 1 m) soil has important implications for mitigating the rise of atmospheric CO_2_ concentration in the future. However, our results showed that soil carbon stocks in restored forests were not significant difference compared with FL, and it just has had slightly positive effects on carbon storage in deep soil. In contrast, there are many reports for carbon sequestration by ecological restoration^5^. To our best knowledge, most previous studies on SOC and SIC with the greatest depths in the Chinese Loess Plateau reported being just 3 m^23,26,30^. Our results indicated substantial soil carbon storages in the deeper soil (e.g. 1–20 m), consistent with the recent findings of Li et al.^11^. Indeed, for the carbon storage evaluation in a deep soil profile like Loess Plateau, estimation should not limit the topsoil layer (e.g. 0–1 to 3 m). Note that deep loessial soils may develop under different vegetation covers at various stages that may have stored SOC historically^17,35^. Li et al.^29^ reported that apple orchard at different stand ages did not significantly increase SOC storage, however, with increased stand age, the coarse root density (> 2 mm), root biomass carbon storage and SWS loss significantly increased in the deep soil profile (below 1 m). This may be explained by root carbon conversion to SOC too little and the root was lignification for extraction water from deep soil strata and the small number of roots in specific layers restricted root-induced SOC change in the living soil layer through fine root turnover and related exudates.

Using a chronosequence approach, this study showed that SOC storage than SIC storage (in contrast) in deep soil is strongly correlated with soil water storage loss (Fig. 7). The predictive relationship between SWS loss in the deep soil and carbon input from root biomass might represent a new avenue for estimating deep carbon storage^29^. To evaluate the response of SWS and soil carbon storage to long-term forest ecological engineering and the coupling interaction between them, we examined the trade-off between carbon accumulation in soil and soil water consumption in the Chinese Loess Plateau. The SWS and SOC storage showed different correlations at different vegetation types and in the different soil layers. Although the conversion of farmland to forest slightly increases SOC storage, it may consume more soil moisture and cause soil desiccation in the Chinese Loess Plateau^46^. In our study, averaged SOC storage of the three restored vegetations was greater by 0.94 kg m^−2^ than FL (*p* > 0.05), which overall consumed 1156.94 mm soil water storage compared with FL (*p* < 0.05), that is, soil accumulation 1 kg m^−2^ SOC need to consume around 1230.80 mm SWS in whole profile (0–20 m).

In the arid and semiarid areas, the consequence of the long-term soil carbon sequestration is at the cost of water depletion and soil desiccation, which positively affects plant productivity in assimilating carbon and contemporarily SOC facilitated soil water retention or wilting point^5,28,47^. Therefore, SWS was positively related to SOC storage in the 1–5 m and 1–10 m layers for different revegetation types. In addition, the SWS was affected by the soil texture, that is, the contents of silt and clay (Fig. S1). However, in the 1–20 m layers this relationship disappears or even becomes negative, which may be explained by lower plant water uptake in the deeper (10–20 m) layers^29^ and precipitation cannot recharge into the deeper soil in the Chinese Loess Plateau^48^. Interestingly, there was no significant correlation between SOC storage and SWS for the FL, but there was a significantly negative correlation between SIC storage and SWS, which was explained by that the SWS is not consumed by crops in deep soil (below 1 m) and it can be replenished by precipitation in FL^11,29^. Besides, our results showed that SWS was positively related to SIC storage in different layers for different vegetation, contrary to Zhao et al.^30^. In this case, the SIC storage below 1 m has a prolonged rate of carbon exchange and has not been influenced by groundwater^40^ and the parent material of this soil is loess, including more inorganic C^38^, revegetation relatively consumes lower soil water content from deeper soil layer. In summary, SOC accumulates through the consumption of soil water by plants and concurrently can increase soil water retention. In our study, afforestation slightly increased SOC storage, but it consumed more SWS from the deep soil profile and resulted in soil desiccation and unsustainable development^29^. In the Chinese Loess Plateau, previous results^48^ have revealed a potential environmental risk related to water availability and C sequestration under afforestation. Based on the dynamics of profile soil moisture and vertical distribution of roots, it was concluded that the depth of depleted soil moisture under PO, PT and AO in the Chinese Loess Plateau could reach 18, 16 and 19 m, respectively^49^. The existence of both negative and positive correlations between soil carbon and water may have reflected short-term cyclic changes with long-term cumulative evolution^28^. For the correlation between soil water and carbon, while the consequence of the long-term soil pedogenic processes is soil carbon formation as a cost of water depletion and soil desiccation, the short-term soil functioning processes is that soil water content of the forestland has a positive effect on plant productivity in assimilating carbon and contemporarily facilitated soil water retention^47^. The present soil-landscape has been shaped by combining long- and short-time processes, and this history can provide some clues to project future changes. Therefore, exploring sustainable afforestation requires adequate soil carbon and soil water in deep soils in arid and semiarid areas. Appropriate plant species selection is also the key to improving water use efficiency and carbon sequestration. If afforestation exceeds the carrying capacity of an ecosystem, degradation is inevitable^22,27^. Water and carbon interaction in deep are significant for the sustainability of ecological restoration in arid and semiarid areas.

## Conclusions

In this study, we highlighted the importance of deep soil in regulating water and carbon cycles, while the “Grain for Green” Project carried out over a large area of the Chinese Loess Plateau may have demonstrated considerable impacts on soil carbon accumulation and soil water balance at the shallow soil depth. Our results showed that under different restored vegetation types, SOC and SIC content and their storage were not significant differences in the whole 20-m soil profile of the semi-arid loess hilly area. The deep loessial soil profile contained a massive amount of inorganic carbon and the SIC storage was 9–10 times SOC storage for the same vegetation. However, restored vegetation types significantly consumed soil water from a deeper soil profile, leading to SWS loss in deep soil. In summary, afforestation did not significantly increase deep soil carbon storage but water depletion. We need to further understand the water-carbon interactions in deep soil for sustainable and eco-environmental construction and regulate water and carbon fixation in a semi-arid region of the Northern Loess Plateau.

## Supplementary Information

Supplementary Information 1.
