# Supplementary material for: Long-term vegetation restoration increases deep soil carbon storage in the Northern Loess Plateau
Source: Sci Rep. 2021 Jul 2;11:13758. doi: 10.1038/s41598-021-93157-0 (PMC8253830; doi:10.1038/s41598-021-93157-0)
Supplement: Supplementary file 1 — Supplementary Information 1. [file 41598_2021_93157_MOESM1_ESM.docx]

Supplementary material for the article entitled: “Long-term vegetation restoration increases deep soil carbon storage in the Northern Loess Plateau” by Lan et al.

Table S1. SOC content of different soil layers for different vegetation types

| Depth (m) | SOC (g/kg) | | | |
| --- | --- | --- | --- | --- |
|  | FL | PO | PT | AO |
| 0.00 | 4.11±1.04a | 5.08±2.79a | 3.32±0.11a | 3.73±0.12a |
| 0.50 | 1.72±0.37a | 1.51±0.21a | 2.26±0.07a | 1.89±0.77a |
| 1.00 | 1.45±0.00a | 1.40±0.37a | 1.82±0.62a | 1.44±0.21a |
| 1.50 | 1.68±0.41a | 1.40±0.02a | 1.82±0.23a | 1.43±0.04a |
| 2.00 | 1.20±0.39a | 1.41±0.15a | 1.79±0.54a | 1.31±0.18a |
| 2.50 | 0.96±0.65a | 1.51±0.34a | 1.70±0.26a | 1.36±0.17a |
| 3.00 | 1.42±0.05a | 1.63±0.31a | 1.73±0.49a | 1.39±0.15a |
| 3.50 | 1.17±0.22a | 1.60±0.34a | 1.61±0.40a | 1.33±0.40a |
| 4.00 | 1.37±0.11a | 1.50±0.23a | 1.88±0.13a | 1.39±0.57a |
| 4.50 | 0.95±0.80b | 1.48±0.06ab | 2.41±0.25a | 1.54±0.54ab |
| 5.00 | 1.47±0.14a | 1.39±0.32a | 1.45±0.62a | 1.84±0.78a |
| 5.50 | 1.35±0.10a | 1.35±0.31a | 1.60±0.73a | 1.73±1.00a |
| 6.00 | 1.25±0.39a | 1.27±0.29a | 1.30±0.17a | 1.38±0.52a |
| 6.60 | 1.26±0.30a | 1.57±0.26a | 1.32±0.67a | 1.29±0.44a |
| 7.00 | 1.20±0.38a | 1.56±0.01a | 1.36±0.22a | 1.36±0.58a |
| 7.50 | 1.30±0.12a | 1.32±0.09a | 1.58±0.18a | 1.70±0.77a |
| 8.00 | 1.20±0.45a | 1.11±0.00a | 1.19±0.26a | 1.52±0.56a |
| 8.50 | 1.22±0.10a | 1.12±0.24a | 1.25±0.11a | 1.42±0.37a |
| 9.00 | 1.23±0.35a | 1.11±0.32a | 1.33±0.00a | 1.22±0.15a |
| 9.50 | 0.92±0.52a | 1.18±0.02a | 1.40±0.22a | 1.34±0.18a |
| 10.00 | 1.14±0.34a | 1.32±0.15a | 1.44±0.24a | 1.31±0.00a |
| 10.50 | 1.14±0.14a | 1.57±0.27a | 1.42±0.59a | 1.38±0.10a |
| 11.00 | 1.09±0.08b | 1.49±0.25ab | 1.49±0.11ab | 1.56±0.11a |
| 11.50 | 1.26±0.16a | 1.39±0.06a | 1.87±0.74a | 1.45±0.22a |
| 12.00 | 1.26±0.10a | 1.49±0.21a | 1.45±0.68a | 1.41±0.78a |
| 12.50 | 1.27±0.22a | 1.43±0.31a | 1.69±0.70a | 1.17±0.62a |
| 13.00 | 1.15±0.07a | 1.22±0.34a | 1.41±0.69a | 0.85±0.21a |
| 13.50 | 1.00±0.21a | 1.41±0.48a | 1.08±0.03a | 0.80±0.14a |
| 14.00 | 1.08±0.09a | 1.10±0.10a | 1.12±0.01a | 0.88±0.19a |
| 14.50 | 1.16±0.19a | 0.95±0.03a | 1.50±0.69a | 1.17±0.08a |
| 15.00 | 1.11±0.12a | 0.96±0.08a | 1.27±0.23a | 1.17±0.21a |
| 15.50 | 1.00±0.14 | 0.98±0.11 | 1.01±0.26 | 1.11 |
| 16.00 | 0.99±0.24 | 0.93±0.00 | 1.02±0.12 | 0.8 |
| 16.50 | 1.17±0.11 | 0.84±0.01 | 1.12±0.37 | 1.04 |
| 17.00 | 1.25±0.13 | 1.08±0.09 | 1.22±0.16 | 0.92 |
| 17.50 | 1.16±0.12 | 0.95±0.09 | 1.31±0.09 | 0.96 |
| 18.00 | 1.38±0.31 | 1.11 | 1.19±0.26 | 0.93 |
| 18.50 | 1.20±0.13 | 1.16 | 1.13±0.28 | 0.74 |
| 19.00 | 0.96±0.07 | 1.11 | 0.96±0.07 | 0.66 |
| 19.50 | 1.13±0.02 | 1.07 | 1.10±0.27 | 0.92 |
| 20.00 | 1.03±0.18 | 0.97 | 0.99±0.22 | 0.93 |

Note: Different lower-case letters indicate significant differences at P<0.05 among vegetation types within each layer. FL denotes farmland, PO denotes *Platycladus orientalis* (Linn.) Franco, PT denotes *Pinus tabulaeformis* Carr. and AO denotes apple orchard.

Table S2. SIC content of different soil layers for different vegetation types

| Depth (m) | SIC (g/kg) | | | |
| --- | --- | --- | --- | --- |
|  | FL | PT | PO | AO |
| 0.00 | 11.50±1.27a | 12.79±1.27a | 12.92±0.79a | 11.85±0.42a |
| 0.50 | 11.59±1.82a | 12.43±0.15a | 12.75±0.91a | 12.30±1.18a |
| 1.00 | 12.11±0.24a | 12.39±0.27a | 13.38±0.88a | 12.69±1.00a |
| 1.50 | 9.589±2.95a | 7.822±3.62a | 11.57±1.31a | 9.912±2.92a |
| 2.00 | 13.12±1.31a | 13.40±1.34a | 15.08±0.60a | 13.64±1.00a |
| 2.50 | 13.53±1.95a | 12.95±1.18a | 14.07±0.39a | 13.29±0.45a |
| 3.00 | 13.27±1.70a | 13.10±0.30a | 13.12±0.57a | 13.36±0.91a |
| 3.50 | 13.48±0.42a | 13.10±0.79a | 13.85±0.33a | 12.88±0.85a |
| 4.00 | 13.40±0.85a | 13.16±0.21ab | 12.19±0.54ab | 11.76±0.36b |
| 4.50 | 12.67±3.77a | 13.42±0.21a | 13.64±1.49a | 12.60±0.15a |
| 5.00 | 13.20±1.73a | 13.79±2.01a | 14.32±0.82a | 14.52±1.88a |
| 5.50 | 13.94±0.76a | 14.48±0.67a | 15.47±1.52a | 14.97±0.45a |
| 6.00 | 13.44±1.58a | 13.25±1.55a | 12.58±0.60a | 12.39±0.82a |
| 6.60 | 13.23±0.00a | 14.09±0.85a | 13.55±0.21a | 13.64±0.45a |
| 7.00 | 13.03±0.88a | 14.17±0.60a | 12.97±1.15a | 12.15±0.48a |
| 7.50 | 12.79±1.27a | 12.86±1.43a | 12.04±0.88a | 11.98±0.97a |
| 8.00 | 14.28±1.55a | 12.49±0.42ab | 10.83±1.06b | 11.16±0.73b |
| 8.50 | 17.04±1.91a | 13.48±0.73ab | 11.85±0.18b | 13.98±1.91ab |
| 9.00 | 15.38±3.16a | 13.59±0.82a | 12.23±0.48a | 13.12±1.31a |
| 9.50 | 13.70±0.18a | 13.25±0.88a | 12.45±1.09a | 12.47±1.73a |
| 10.00 | 13.25±0.82a | 13.05±0.54a | 11.78±0.15a | 12.75±1.15a |
| 10.50 | 13.16±0.21a | 13.42±0.70a | 12.82±1.79a | 12.67±1.03a |
| 11.00 | 12.45±0.85a | 15.01±0.88a | 15.08±3.47a | 13.48±0.85a |
| 11.50 | 14.17±2.49a | 15.19±0.15a | 15.34±3.59a | 15.64±4.51a |
| 12.00 | 11.01±0.27b | 19.84±4.66a | 14.50±1.85ab | 10.68±0.54b |
| 12.50 | 11.11±0.67a | 18.03±6.06a | 13.51±0.15a | 12.32±1.82a |
| 13.00 | 10.88±1.55a | 16.37±4.32a | 13.79±2.31a | 11.72±2.55a |
| 13.50 | 13.89±4.11a | 14.32±2.83a | 12.54±0.12a | 10.81±1.40a |
| 14.00 | 12.69±2.71a | 15.92±3.99a | 13.74±2.19a | 8.985±1.98a |
| 14.50 | 13.29±0.88a | 14.82±2.19a | 12.88±1.76a | 10.70±2.52a |
| 15.00 | 13.12±3.26a | 10.90±1.46a | 13.10±0.60a | 13.42±1.12a |
| 15.50 | 14.17±3.71 | 13.25±0.63 | 12.99±0.51 | 14.48 |
| 16.00 | 11.37±0.60 | 9.804±1.12 | 12.49±0.06 | 17.84 |
| 16.50 | 12.43±2.77 | 10.06±0.88 | 13.61±1.64 | 16.89 |
| 17.00 | 11.87±0.94 | 13.89±4.35 | 12.82±0.94 | 13.1 |
| 17.50 | 13.31±3.10 | 11.78±1.91 | 13.14±1.70 | 10.98 |
| 18.00 | 13.51±0.33 | 9.61 | 13.12±0.70 | 8.66 |
| 18.50 | 10.81±4.02 | 11.8 | 12.51±0.76 | 10.21 |
| 19.00 | 14.52±0.85 | 13.18 | 12.00±0.82 | 16.59 |
| 19.50 | 15.57±2.16 | 19.86 | 10.73±1.34 | 12.06 |
| 20.00 | 14.04±1.03 | 22.32 | 12.23±1.03 | 17.88 |

Note: Different lower-case letters indicate significant differences at P<0.05 among vegetation types within each layer. FL denotes farmland, PO denotes *Platycladus orientalis* (Linn.) Franco, PT denotes *Pinus tabulaeformis* Carr. and AO denotes apple orchard.

Table S3. SOC, SIC storage and SWS of different soil layers for different vegetation types

|  | Layer/m | FL | PO | PT | AO |
| --- | --- | --- | --- | --- | --- |
| SOC storage | 0-0.5 | 2.55±0.64Aa | 3.08±1.69Aa | 2.05±0.07Aa | 2.17±0.07Aa |
|  | 0.5-1 | 1.02±0.22Ba | 0.9±0.13Ba | 1.43±0.05ABa | 1.09±0.45Aa |
|  | 1-1.5 | 1.02±0.25Ba | 0.84±0.01Ba | 1.12±0.14Ba | 0.85±0.03Ba |
|  | 1.5-2 | 0.72±0.24Ba | 0.86±0.09Ba | 1.18±0.36Ba | 0.78±0.11Ba |
| SIC storage | 0-0.5 | 7.12±0.79Aa | 7.75±0.77Aa | 7.98±0.49Ba | 6.87±0.25Aa |
|  | 0.5-1 | 6.84±1.08Aa | 7.37±0.09Aa | 8.05±0.58Ba | 7.07±0.68Aa |
|  | 1-1.5 | 5.79±1.79Aa | 4.66±2.16Aa | 7.07±0.8Ba | 5.85±1.73Aa |
|  | 1.5-2 | 7.89±0.79Ab | 8.08±0.81Aab | 9.97±0.4Aa | 8.12±0.6Aab |
| SWS | 0-0.5 | 5.89±1.72Aa | 4.54±3.66Aa | 7.45±0.17Aa | 3.06±0.68Aa |
|  | 0.5-1 | 5.16±0.64Aa | 3.39±1.07Aa | 4.84±2.81Aa | 2.27±0.66Aa |
|  | 1-1.5 | 5.46±0.87Aa | 2.54±0.62Ab | 3.47±0.05Ab | 2.81±0.76Ab |
|  | 1.5-2 | 6.32±0.58Aa | 2.65±0.03Ac | 3.82±0.27Ab | 2.86±0.4Abc |

Note: FL denotes farmland, PO denotes *Platycladus orientalis* (Linn.) Franco, PT denotes *Pinus tabulaeformis* Carr. and AO denotes apple orchard. Different upper-case letters indicate significant differences at P<0.05 among different soil layer. Different lower-case letters indicate significant differences at P<0.05 among vegetation types within each layer.


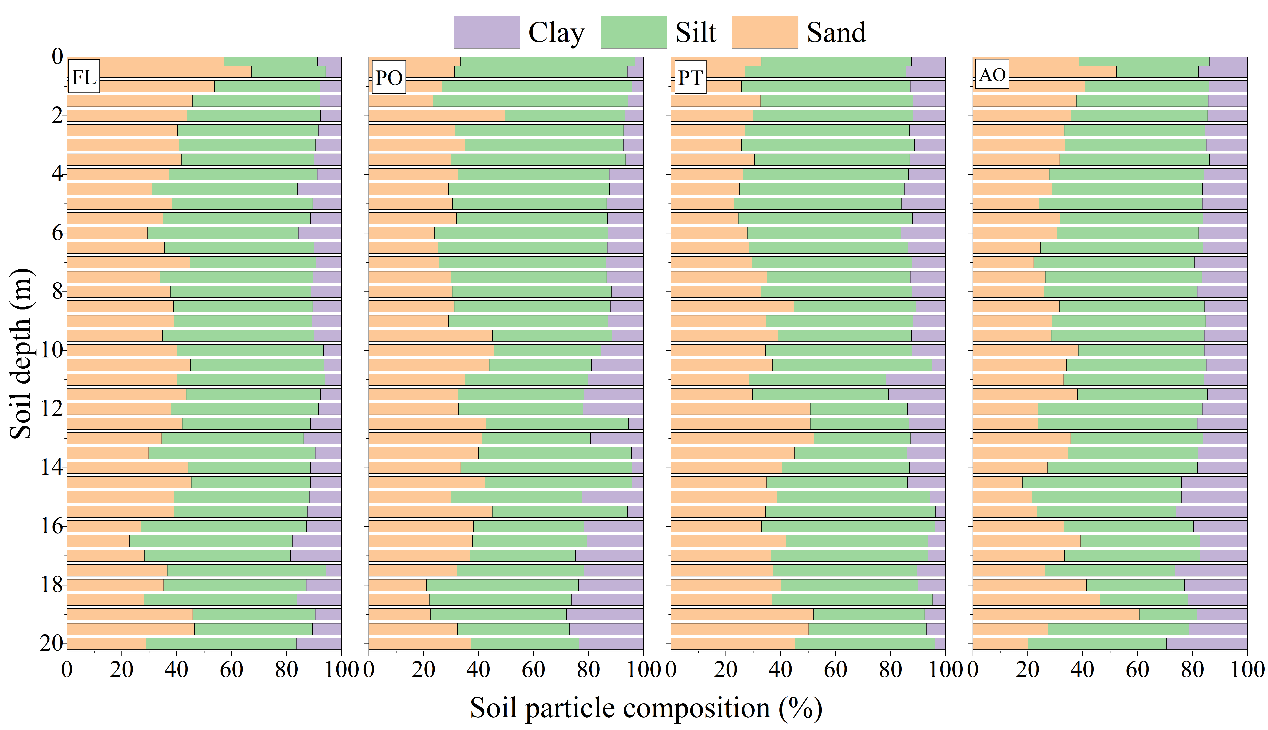


Figure S1 The vertical distribution of soil particle composition under different vegetation types. Note: FL denotes farmland, PO denotes Platycladus orientalis (Linn.) Franco, PT denotes Pinus tabulaeformis Carr. and AO denotes apple orchard.
